# Queueing theory model of pentose phosphate pathway

**DOI:** 10.1038/s41598-022-08463-y

**Published:** 2022-03-17

**Authors:** Sylwester M. Kloska, Krzysztof Pałczyński, Tomasz Marciniak, Tomasz Talaśka, Marissa Miller, Beata J. Wysocki, Paul Davis, Tadeusz A. Wysocki

**Affiliations:** 1 Faculty of Medicine, Nicolaus Copernicus University Ludwik Rydygier Collegium Medicum, 85-094 Bydgoszcz, Poland; 2 Faculty of Telecommunications, Computer Science and Electrical Engineering, Bydgoszcz University of Science and Technology, 85-796 Bydgoszcz, Poland; 3 Department of Electrical and Computer Engineering, University of Nebraska-Lincoln, Omaha, NE 68182 USA; 4 Department of Biology, University of Nebraska at Omaha, Omaha, NE 68182 USA

**Keywords:** Biological models, Metabolomics, Carbohydrates, Enzyme mechanisms, Enzymes, Metabolomics, Cell growth, Biochemical reaction networks, Cellular signalling networks, Computational models, Computational platforms and environments, Machine learning, Statistical methods, Biochemical networks, Computer modelling, Dynamic networks, Dynamical systems, Metabolic engineering, Stochastic modelling, Stochastic networks, Biochemistry, Cell biology, Computational biology and bioinformatics, Systems biology

## Abstract

Due to its role in maintaining the proper functioning of the cell, the pentose phosphate pathway (PPP) is one of the most important metabolic pathways. It is responsible for regulating the concentration of simple sugars and provides precursors for the synthesis of amino acids and nucleotides. In addition, it plays a critical role in maintaining an adequate level of NADPH, which is necessary for the cell to fight oxidative stress. These reasons prompted the authors to develop a computational model, based on queueing theory, capable of simulating changes in PPP metabolites’ concentrations. The model has been validated with empirical data from tumor cells. The obtained results prove the stability and accuracy of the model. By applying queueing theory, this model can be further expanded to include successive metabolic pathways. The use of the model may accelerate research on new drugs, reduce drug costs, and reduce the reliance on laboratory animals necessary for this type of research on which new methods are tested.

## Introduction

In recent years, there has been significant progress in metabolomics. New and improved test methods allow for the measurement of many important biochemical parameters. The acquired data can be used to create simulation models of biochemical reactions and entire metabolic pathways. Queueing theory can successfully model metabolic processes, as demonstrated by the example of the glycolysis pathway^1^ and Krebs cycle^2^. The preparation of an accurate model simulating the course of PPP could potentially reduce the time needed for drug testing and reduce the number of laboratory animals on which new drugs are tested^3^.

The PPP is a metabolic pathway whose main substrate is glucose-6-phosphate (G6P). Throughout the reactions that make up this pathway, numerous molecules are formed, such as: nicotinamide adenine dinucleotide phosphate (NADPH), which is used in the biosynthesis of fatty acids, ribose 5-phosphate (R5P), which is a precursor in the synthesis of nucleotides, and erythrose 4-phosphate (E4P), which is used in the synthesis of aromatic amino acids^4,5^. Products of the PPP are essential for the formation of new cells. However, under stress, cell growth is slows down and the PPP is responsible for maintaining cellular levels of NADPH. In fact, such conditions increase the reliance of the PPP in the cell over glycolysis to maintain the needed ratio between NADP$^{+}$ and NADPH^6^. In most living organisms, this pathway takes place in the cell cytosol.

There are two phases in the PPP: the oxidative phase and the non-oxidative phase. During the oxidative phase, NADPH is produced^7^. In the non-oxidative phase, various simple sugars are synthesized. 5-carbon sugars derived from the digestion of nucleic acids can be utilized in the PPP, where their carbon backbones are metabolized into intermediates for glycolysis or gluconeogenesis. In the non-oxidative phase, one of the enzymes- transketolase—is responsible for catalyzing two different reactions, with two sets of substrates. Therefore, these substrates act as inhibitors to each other, since they are competing for the same active site of the enzyme.

It is estimated that as much as 60% of NADPH comes from the PPP^8^. The PPP is most active in the liver, adrenal cortex, and mammary glands. The activity for this pathway is high in red blood cells, making it extremely important in erythrocytes^9^. NADPH formed by the PPP is used in the cell to prevent oxidative stress and the formation of dangerous free radicals that could harm the cell^10^. Reactive oxygen species (ROS) can damage cellular lipids, proteins, and nucleic acids, and eventually cause cell death^11^. It is worth noting that ROS are associated with many diseases^12–14^. Since erythrocytes do not have mitochondria, they have no other source of reducing oxidative stress other than the PPP. For example, large amounts of NADPH generated in erythrocytes are used to reduce glutathione (GSH). This reduced form of GSH is essential for maintaining the proper state of the cell. If GSH level is lowered in erythrocytes, hemolysis may occur^15^.

The most common approach used to model metabolic changes in a cell is to use Ordinary Differential Equations (ODE). For metabolic reactions, ODEs provide quantitative information on interactions that occur between metabolites in specific reactions taking place in the cell. Previously, ODEs have been successfully used in simulation studies of biochemical kinetics and biochemical connections^16–18^. The authors in^19^ presented a PPP model based on ODEs. This approach was beneficial because it did not require complicated operations that strained the capabilities of computers in the past, resulting in lower computing power. However, the simplifications and assumptions made when using ODEs in metabolic simulations do not reflect the stochastic nature of cell biochemistry^20^. The Chemical Master Equation (CME) was another approach used to model the stochasticity of biological reactions^21^. However, due to the complexity and computing requirements, networks based on these models cannot be too extensive. A relatively new approach to computational metabolic modeling is the use of queueing theory. Queueing theory has wide applications in telecommunications, but also in biological and medical science topics, such as modeling drug pharmacokinetics^22^ or HIV infectivity^23^. Using this method, it was possible to accurately model a simple metabolic network and mimic the interactions between metabolites^24^, as well as the Krebs cycle^2^. A genetic algorithm was used to optimize the kinetic coefficients. A variety of AI methods can be used for this purpose, but genetic algorithm was chosen because it was used with success when modeling the Krebs cycle.

The aim of this work was to prepare a PPP model capable of tracking concentration changes of specific metabolites occurring in this pathway over time. Additionally, the usefulness of the genetic algorithm for finding values of the kinetic constants used in the model was confirmed^2^. A genetic algorithm was used to find values corresponding to those in the literature.

## Results

**Figure 1:**
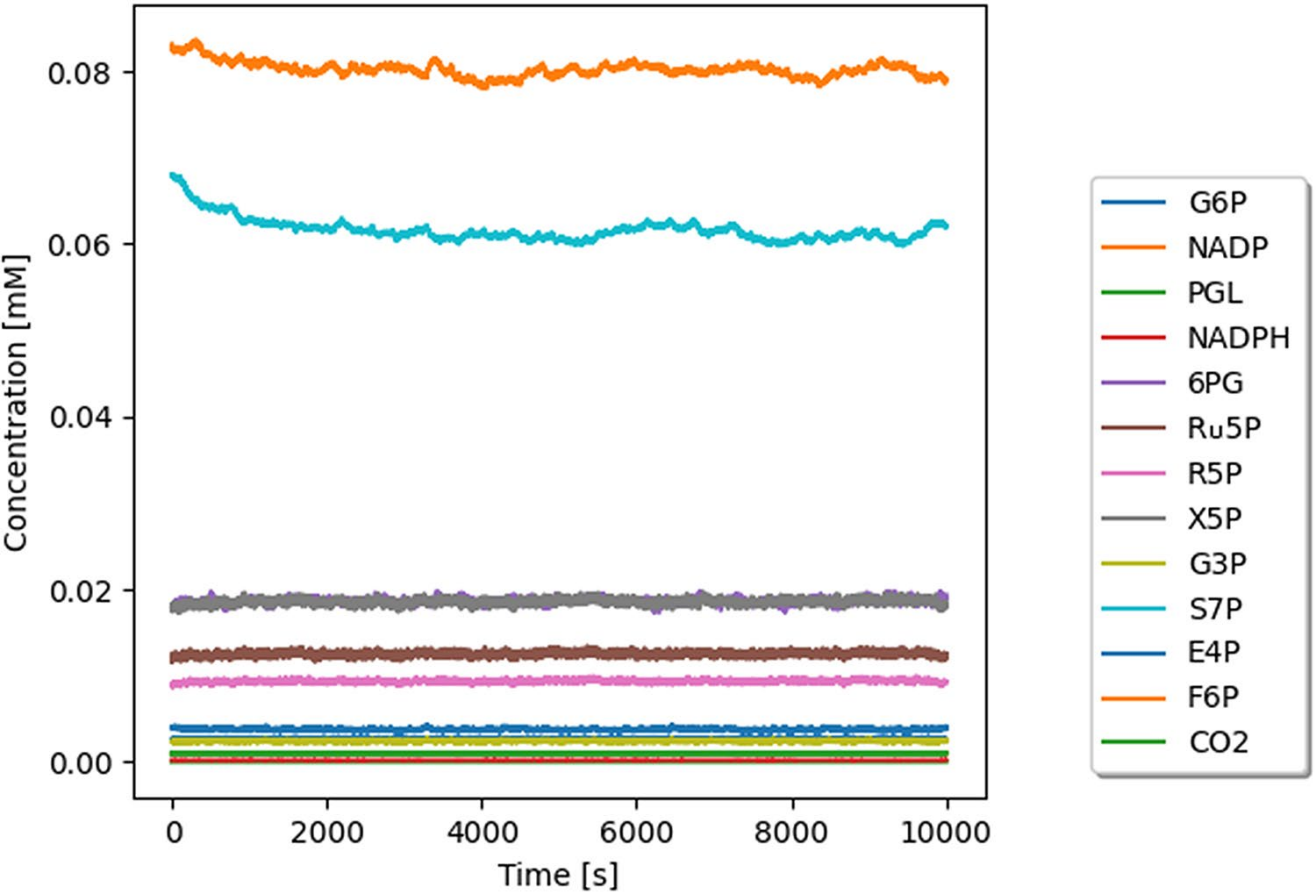
Concentration level change over time under unperturbed conditions. *G6P* glucose-6-phosphate, *NADP* NADP+, *PGL* 6-P-gluconolactone, *6PG* 6-phosphogluconate, *Ru5P* ribulose-5-phosphate, *R5P* ribose-5-phosphate, *X5P* xylulose-5-phosphate, *G3P* glyceraldehyde-3-phosphate, *S7P* sedoheptulose-7-phosphate, *E4P* erythrose-4-phosphate, *F6P* fructose-6-phosphate.

**Figure 2:**
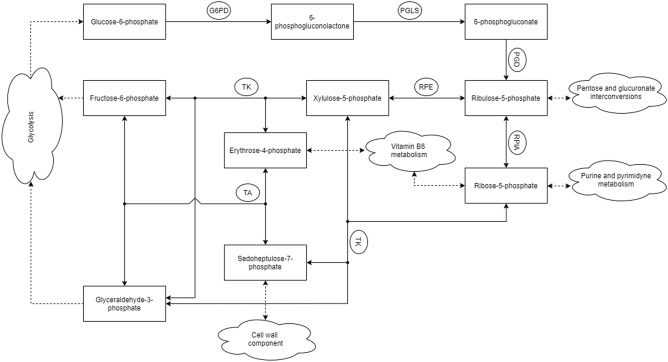
PPP scheme; the graph shows the main carbohydrate products, their relations with other metabolic pathways, and enzymes that catalyze reactions. *G6PD* glucose-6-phosphate dehydrogenase, *PGLS* 6-phosphogluconolactonase, *PGD* 6-phosphogluconate dehydrogenase, *RPIA* ribose-5-phosphate isomerase A, *RPE* ribulose-5-phosphate-3-epimerase, *TA* transaldolase, *TK* transketolase.

The generated model becomes stable within approximately one hour. Every second, there are 1000 simulations of each pathway reaction (or 1 simulation step per millisecond), averaged over 50 simulated cells. This number has been selected experimentally. However, the model is designed to vary this number depending on the needs of the researcher. Figure 1 shows concentration changes of individual metabolites over time. Due to the various conditions of the living cell, G6P and NADP are consumed faster or slower depending on the blood glucose level, since glucose is phosphorylated to G6P to stay inside the cell and prevent diffusion out of the cell. This affects the glucose level in the cell, so the variation of 10% was assumed. The variation level is an arbitrary choice; meaning it can be changed. The purpose for the use of variation is to represent the concentration fluctuations in the cell. For this model to reflect the flow of metabolites in the cell as accurately as possible, the so-called “balancing flow” was used^1,2^. This feature allows for proper simulation of metabolite flow depending on the current needs of the cell (Fig. 2). Thus, the level of metabolites that occur in more than one metabolic pathway, e.g. F6P and G3P being part of the PPP and glycolysis, better mimics biological conditions. Table 1 presents the comparison of model generated data and literature data regarding concentration of individual metabolites.

**Table 1:** Comparison of concentration data: literature and model (mmol/L). Calculated relative difference shows similarity of obtained results and literature data.

Metabolite	Initial conc. (literature)	Final conc. (model)	Standard deviation over mean (%)	Absolute difference	Relative difference (%)
Glucose-6-P (G6P)	0.0026	0.0026	3	0	0
NADP$^{+}$	0.001	0.001	3	0	0
NADPH	0.0002	0.0002	3	0	0
6-P-gluconolactone (PGL)	$5 \times 10^{-6}$	$9.3 \times 10^{-6}$	36	$4.3 \times 10^{-6}$	86
6-P-gluconate (6PG)	0.018	0.019	2	0.001	5.5
Ribulose-5-P (Ru5P)	0.012	0.012	2	0	0
Ribose-5-P (R5P)	0.009	0.009	1	0	0
Xylulose-5-P (X5P)	0.018	0.018	1	0	0
Glyceraldehyde-3-P (G3P)	0.00234	0.00242	3	0.00008	3.4
Sedoheptulose-7-P (S7P)	0.068	0.062	1	0.006	8.8
Erythrose-4-P (E4P)	0.004	0.004	3	0	0
Fructose-6-P (F6P)	0.083	0.079	0	0.004	4.8

PGL is rapidly hydrolyzed, so the practical equilibrium between G6P and 6PG is directed towards the formation of 6PG^25^. Any existing PGL is almost immediately converted to 6PG, therefore the variance is very high. The relative difference of PGL is high because it depends on the measurement time. In the future, we intend to combine the PPP with the already developed Krebs cycle and glycolysis models, so the results of the PPP model are likely to be closer to the experimental results.

Due to the high demand of glucose and its metabolites by cancer cells, many drugs are aimed at blocking metabolic pathways that supply cancer cells with substances necessary for proliferation. The PPP is one of the pathways with significantly increased activity in neoplastic cells. Compared to healthy cells, the activity of the PPP in cancer cells can be increased up to 8 times. The oxidative part of the pathway provides cells with a large amount of NADPH, helping the cell can effectively fight excess oxidative stress. Effects that reduce the effectiveness of the production of NADPH in the cell, in combination with factors that induce this stress, such as radiotherapy or chemotherapy, can kill cancer cells.

**Figure 3:**
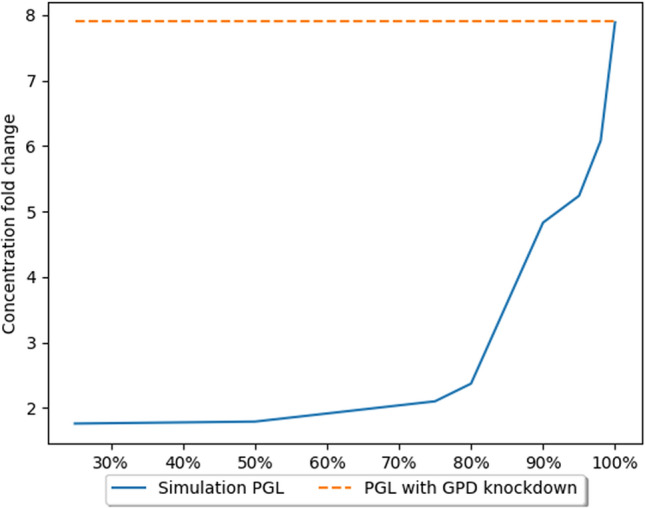
The effects of GPD gene expression knockdown on PGL concentration^26^. The X axis presents level of simulated GPD inhibition. The Y axis presents fold change in concentration in comparison to the natural state (without inhibition).

**Figure 4:**
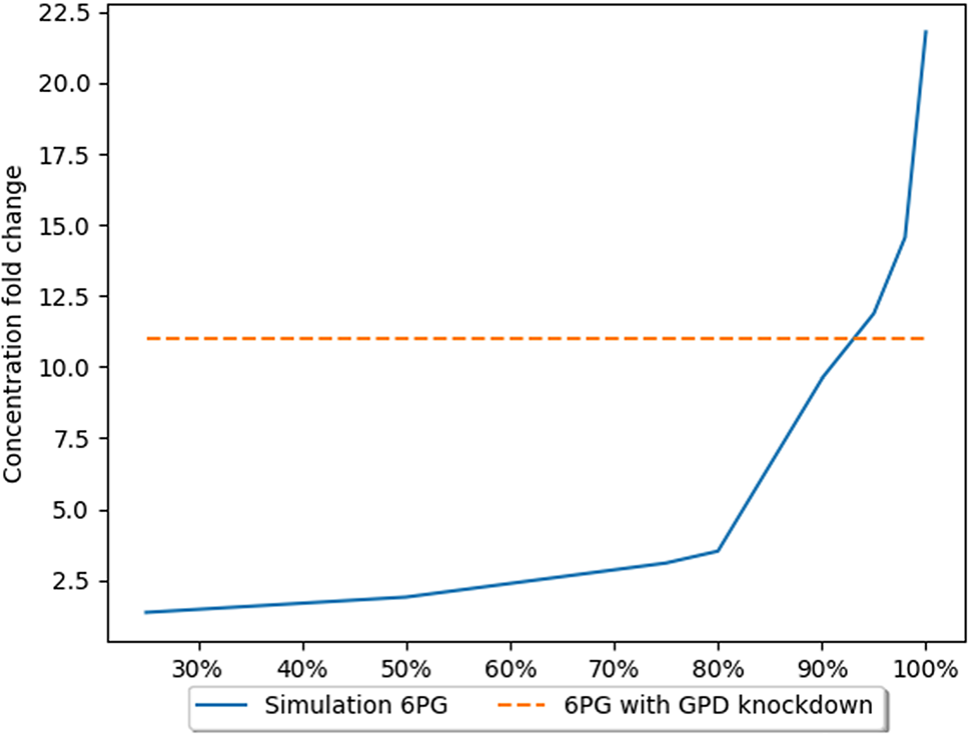
The effects of GPD gene expression knockdown on 6PG concentration^26^. The X axis presents level of simulated GPD inhibition. The Y axis presents fold change in concentration in comparison to the natural state (without inhibition).

To validate the model, model results were compared to those obtained empirically. The paper^26^ serving as the benchmark for our model described the effect of a third PPP enzyme, PGD, in lung cancer cells. Inhibition of this enzyme’s activity does not significantly affect the level of NADPH, but inhibits tumor growth. The gene encoding PGD is characterized by increased expression in neoplastic cells. ShRNA molecules were used to reduce PGD expression. This approach resulted in inhibition of tumor growth , indicating an important role for PGD in cancer cell metabolism. Concentrations of several PPP metabolites were measured, however, not all of them had significant changes. Metabolites of the oxidative phase of the PPP, such as 6-phosphogluconolactone (PGL) and 6-phosphogluconate (6PG) had concentrations 7.9 and 11 times higher than their regular concentrations, respectively (Figs. 3 and 4). These metabolites accumulated due to the absence/decreased activity of PGD. The concentrations of metabolites of the non-oxidative phase of the pathway such as S7P or X5P were not measured, but no significant changes in the concentrations of ribose phosphate and nucleotide triphosphate were detected.

**Figure 5:**
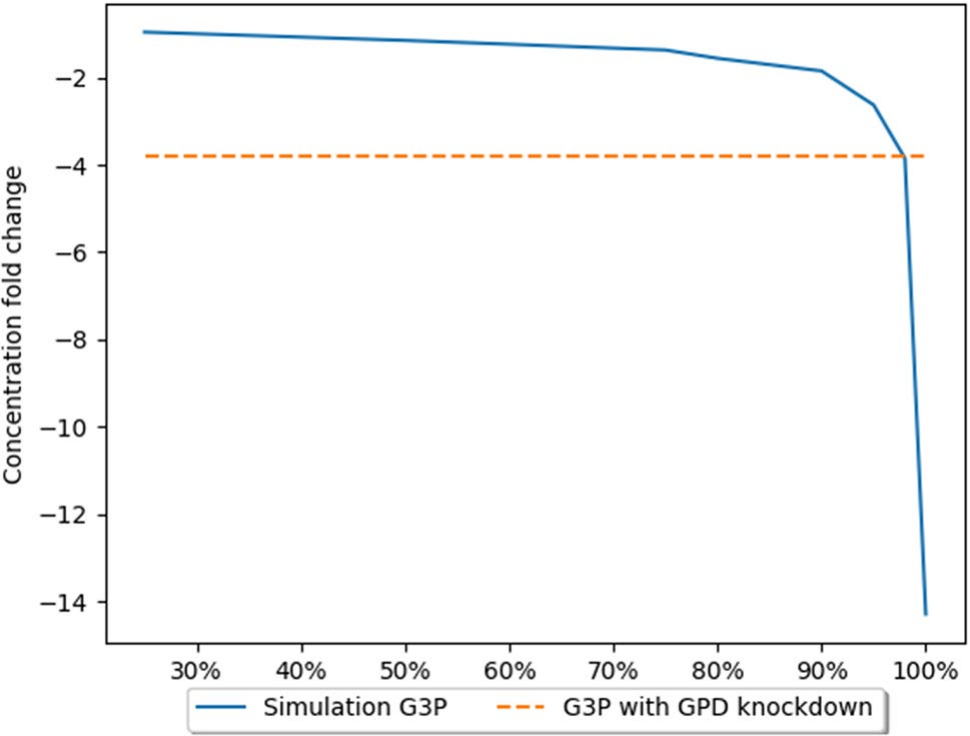
The effects of GPD gene expression knockdown on G3P concentration^26^. The X axis presents level of simulated GPD inhibition. The Y axis presents fold change in concentration in comparison to the natural state (without inhibition).

The accumulation of metabolites preceding the blocked reaction is because the expression of the PGD enzyme has been reduced. A bottleneck is created at this stage of the pathway, leading to a reduced efficiency of this stage, as there are not enough protein molecules in the cell to process all metabolite molecules. As a further consequence, a decrease in the concentration of metabolites occurring further down the pathway, e.g., G3P, can be observed (Fig. 5). For the validation of the model, measurements of the concentrations of metabolites obtained empirically were used. The model makes it possible to simulate and track the changes in the concentrations of the metabolites.

Several measurements were performed to evaluate the level of inhibition of the GPD catalyzed reaction. The obtained results show that the GPD knockdown caused inhibition at the level of 95–98%. These assumptions are based on the results presented in Table 2. The results for these inhibition levels are the closest to the empirical results. The paper^26^ used shRNA to achieve expression knockdown, which is an incomplete mechanism to reduce (but not eliminate) expression. This form of knockdown is not expected to achieve 100% silencing. Indeed, 80–99% knockdown of expression is normal and expected. The calculated results are comparable to those obtained experimentally and are consistent with current biological knowledge. Another point to consider is that the glucose metabolism of neoplastic cells remains unknown in some aspects and these cells may possibly bypass a blocked reaction in the metabolic pathway. Simulations using 100% inhibition were also performed, but this led to a significant reduction in the concentration of metabolites downstream of the bottleneck of the pathway. However, it can be observed that due to the bidirectional character of reactions of the second phase of the PPP and the flux of metabolites from other pathways, e.g., F6P generated in glycolysis, we do not observe a complete ‘zeroing’ of metabolite concentration.

**Table 2:** Comparison of metabolite concentration changes (fold changes) caused by knockdown of the PGD gene.

Metabolite	Experimental data concentration change^26^	Model data concentration change using 90% inhibition	Model data concentration change using 95% inhibition	Model data concentration change using 98% inhibition	Model data concentration change using 100% inhibition
G6P	1.8	1.8	1.8	1.8	1.8
PGL	7.9	4.83	5.24	6.08	7.89
6PG	1	9.59	11.88	14.56	21.8
G3P	− 3.8	− 1.85	− 2.63	− 3.85	− 14.29

The results generated in our model (Table 2) follows the trend of changes in concentration observed in vitro, and suggests that knockdown efficiency in vitro was likely near 95%, which is common for shRNA expression knockdown.

## Discussion

As mentioned in the introduction, most previous models simulating metabolic pathways, not only PPP, have been based on the use of ODEs. However, due to the advantages offered by queueing theory, it seems reasonable to use this method in modeling. The preparation of a quantitative model of a biological pathway such as the PPP requires the necessary information on starting concentrations and kinetic data of the enzymes that catalyze the pathway reactions. The presented model can be viewed as a ’virtual laboratory’. This model tracks the relationships between individual metabolites formed at different stages of the pathway. It is possible to observe changes caused by fluctuations in metabolite concentrations and their impact on the entire pathway.

It can also be used to test the effectiveness of new drugs if their influence on the kinetics of the reaction they affect is known. In this way, one can also theoretically get answers to questions such as which reactions are worth blocking to obtain the best possible therapeutic result. Most studies aimed at blocking the PPP pathway in cancer patients have focused on blocking the first reaction of the pathway catalyzed by G6PD^27,28^. However, clinical data indicate that this therapy is not very effective without additional exposure to oxidative stress^29,30^. For this reason, the results of studies on the knockdown of the gene encoding PGD in this paper were used^26^. Even though the knockdown of the G6PD gene does not affect the amount of NADPH, which is important for tumor development, the knockdown of this gene alone results in inhibition of tumor growth. Perhaps the metabolites that accumulate in the cell prior to the blocked reaction are responsible for this situation. Their concentration in cells reaches values significantly greater than their natural concentrations. The exact mechanism of tumor growth inhibition is unknown, however, the effect achieved is important.

The proposed model obtained stability based on the data from the above-mentioned paper. We believe that this type of model can be used to predict the impact of therapy, which in turn will lead to an increase in its effectiveness.

Thanks to the use of experimental data together with a computational process based on the queueing theory, a model was obtained that can track the metabolic pathway that takes place in the cells of living organisms. In this paper, we present a separate PPP model without detailed analysis of the relationship between PPP and glycolysis. The metabolites common to both pathways have been identified and several principles have been adopted to create a functional PPP model. In the future, our plan is to connect the existing glycolysis, Krebs cycle, and PPP models together. We believe that such a procedure may also positively affect the consistency of simulation and experimental results.

The presented results indicate that the model can be used to predict changes in metabolite concentrations. For this purpose, it is sufficient to enter the concentration value of one of the metabolites. In this way, the entire study can prove to be more cost-effective—no need to determine each metabolite separately, which also saves time.

As demonstrated by the knockdown of one of the genes encoding the enzyme catalyzing the PPP reaction, this model is adapted to follow the trend of metabolite changes. Moreover, it can determine the specific effect of the inhibition of particular reactions on the concentration of metabolites with relatively high accuracy. Further research providing data on how inhibition of a particular pathway step may affect kinetic constants could contribute to an increase in the accuracy of the presented model.

## Methods

### Obtaining metabolic data and the use of enzymatic reaction kinetics

This work focuses on the reflection of changes in PPP metabolite concentrations over time. For this purpose, a literature review was carried out to provide data on these concentrations (Table 1). Presented concentrations were measured with the use of mass spectrometry^31^. Several kinetic constants, and enzymatic properties, like maximum velocity ($V_{max}$), necessary for the correct operation of the model were used to calculate the speed of chemical reactions^31^. Reaction rates were calculated using equations based on Michaelis–Menten kinetics (for more information please check Supplementary Information).

NADPH is formed from 2 NADP$^{+}$ molecules in the oxidative phase. The energy generated during the conversion of G6P into ribulose 5-phosphate (Ru5P) is used in the reaction. The overall reaction of the first phase of the pathway is as follows:

$$G6P + 2 NADP^{+} + H_{2}O \xrightarrow {} Ru5P + 2 NADPH + 2 H^{+} + CO_{2}$$

Ru5P, which is one of the products of the first phase of the PPP, is the first substrate for the non-oxidative phase. Ribose-5-phosphate isomerase can convert Ru5P to R5P. On the other hand, ribulose 5-phosphate epimerase converts Ru5P to xylulose 5-phosphate (X5P). The next reactions involve changing the length of the carbon chain in the carbohydrates. These two five-carbon sugars then undergo a transketolase-catalyzed reaction. The result is production of glyceraldehyde 3-phosphate (G3P) and sedoheptulose 7-phosphate (S7P). Then G3P and S7P undergo a transaldolase-catalyzed reaction, which produces E4P and fructose 6-phosphate (F6P) (Fig. 2; Table 3).

**Table 3:** Stoichiometric reactions of the PPP. Reactions 1-3 form the oxidative branch of PPP, reactions 4-7 are in the non-oxidative branch.

Number	Reaction	Enzyme
1	$G6P + NADP^+ \xrightarrow {} PGL + NADPH+ H^+$	Glucose 6-phosphate dehydrogenase
2	$PGL + H_{2}O \xrightarrow {} 6PG + H^{+}$	6-Phosphogluconolactonase
3	$6PG + NADP^{+} \xrightarrow {} Ru5P + NADPH+ H^+ + CO_{2}$	6-Phosphogluconate dehydrogenase
4A	$Ru5P \xrightarrow {} R5P$	Ribose-5-phosphate isomerase
4B	$Ru5P \xrightarrow {} X5P$	Ribulose 5-phosphate 3-epimerase
5	$R5P + X5P \xrightarrow {} G3P + S7P$	Transketolase
6	$X5P + E4P \xrightarrow {} G3P + F6P$	Transketolase
7	$G3P + S7P \xrightarrow {} E4P + F6P$	Transaldolase

### Queueing theory

The complicated nature of metabolic pathways, in which there are huge amounts of biochemical substances constituting the substrates and reaction products, makes modeling metabolism extremely challenging. Methods commonly used to model metabolic pathways require supervision and the use of appropriate constraints, like forcing ODEs not to reach negative values. Such treatments may cause small calculation errors which could accumulate in long-term modeling and result in incorrect calculations. Biological systems are organized to pass the products of individual metabolic reactions further down the pathway, so that they become substrates for downstream reactions or are used by the cell to support necessary life processes^32^. For this reason, the use of queueing theory in metabolic pathway modeling seems to be the right approach.

Queueing networks can be thought of as hidden Markov chains, similar to Gillespie’s modelling technique^20,21^. The advantage of using queueing theory to model metabolic pathways is that they do not require enhanced computing power. Therefore, the results can be obtained close to real time. Networks based on queueing theory can be applied with ease to a significantly greater number of molecules, grouped into the queues representing different molecular species. Due to the nature of this approach, it is capable of combining individual pathways into larger, more complex groups of metabolic pathways.

Averaging the results from several simulation runs provides information on the average changes in the concentrations of the individual pathway metabolites. The proposed model is based on calculations of the kinetics of Michaelis–Menten enzymatic reactions, which focus on the relationship between the concentrations of the substrate and the product, and the velocity of the reaction. According to this theory, the macroscopic concept of enzymatic reaction speed is the sum of many microscopic reactions that can exchange specific amounts of substances per time unit. The description of the PPP as the probability of decreasing and increasing the concentration of each of the substances present in the pathway and the correlation of their reduction with the accumulation of other substrates results in a self-regulating, stochastic process that imitates the actual course of the PPP. The Michaelis–Menten kinetic equation was used to calculate the probability of the reaction. A detailed description of the methodology used is described in the work describing the Krebs cycle model^2^.

The probability of the reaction can be converted to an average amount of arrivals when measured for a significant amount of time. Therefore, the kinetic equations can be used to calculate the adaptive parameter $\mu (t)$ utilized for modelling PPP behavior by a network of inhomogeneous Poisson processes described by equation (1):

1 $$\begin{aligned} \begin{aligned} P[(N(t+\tau )-N(t))=k, t]=\frac{e^{-\mu (t)\tau }(\mu (t)\tau )^{k}}{k!} \end{aligned} \end{aligned}$$

Where:

- $P{(N(t+\tau )-N(t))=k, t]}$—probability of *k* arrivals in the interval $(t,t+\tau )$
- $\mu (t)\tau$—expected number of arrivals in a time interval duration of $(t,t+\tau )$

The queue processing time of metabolite increment is described by the exponential distribution of the random variable *T* in the terms of the rate parameter $\mu (t)$ as follows (2):

2 $$\begin{aligned} \begin{aligned} f(T;\mu (t)) = \left\{ \begin{array}{ll} \mu (t) e^{-\mu (t) T} &{}\quad \text {when }T \ge 0\\ 0 &{}\quad \text {when }dT<0\\ \end{array} \right. \end{aligned} \end{aligned}$$

Therefore, the PPP is modelled by the composition of interconnected queues. Departure of substrate’s increment from one queue is followed by the arrival at the successive queue. It is worth noting that the network of interconnected queues is equivalent to the set of ODEs as proven by^33^.

Probability of substrate’s increment departure from each queue depends on the current concentration of the substrates and the kinetic constants of the reaction causing that departure. Every queue uses its individual Michaelis–Menten kinetic equation with kinetic constants normalized according to the method based on the formula described in^1^, to determine the likelihood that in this time step the reaction occurs. Since the reaction rates depend on the current concentration of molecules that change from step to step, the resulting inhomogeneous Poisson process implements the feedback loop, which results in a system with memory.

### Use of a genetic algorithm to optimize model parameters

Values of enzyme kinetic constants were found with the use of a genetic algorithm starting from literature data. Every ‘gene’ in the ‘chromosome’ is a vector of kinetic constants describing each Michaelis–Menten kinetic equation. The new values of kinetic constants are found by randomly selecting from which ‘parent’ ‘offspring’ inherits ‘gene’ (set of kinetic constants for a particular reaction). However, mutation occurs on each kinetic value regardless to which parent it belongs. The loss function optimized by the algorithm is the sum of the squared distances between PPP state described by the literature and the current optimization step of the simulation using kinetic constants that makes an individual ‘chromosome’. The formula of loss function is as follows (3):

3 $$\begin{aligned} \begin{aligned} f(X_{l};X)=\sum (X_{l}-X)^{2} \end{aligned} \end{aligned}$$

Where: $X_{l}$—vector of substrates described by a literature; *X*—vector of substrates describing stable state of simulation.

The loss function described above has a trivial solution. If all kinetic constants that are used in Michaelis–Menten reactions as multipliers (instead of dividers) are zeroed, then the results of these equations are equal to zero. As a result, no reactions occur, so the simulation’s stable point is equal to the original literature vector. To prevent such a solution, the genetic algorithm sets a constraint on newly generated ‘chromosomes’. Each reaction parametrized by values of the ‘chromosome’ for a literature vector of substrates must have a probability of occurrence between 0.00005 and 0.05.

The first set of ‘chromosomes’ are made of Michaelis–Menten kinetic constants defined in the literature with added gaussian noise. Given the selected starting point, the genetic algorithm is set on finding the optimal value in the proximity of the already established values. This reduces the risk of the algorithm generating an output that minimizes the loss function, but produces kinetic constants significantly different from the literature values.

### Pseudocode of the PPP model

The pseudocode describing the computational processes can be found in the Supplementary Information. This code assumes that:

- Kinetic constants are grouped into a table of vectors of constant values. There are 14 vectors in the table corresponding to eight different reactions and six balancing flows. Each of the reactions has a unique vector of dimension equal to the number of kinetic constants used in the reaction rate computation and every balancing flow contains a one-dimensional vector.
- Concentration increment exchanged during the reactions is denoted ‘delta’ and is unique for each reaction. It ranges from $2.3 \times 10^{-6}$ mM to $5.0 \times 10^{-5}$ mM. ’Delta’ is significantly lower than the initial value of the lowest substrate concentration. The ‘delta’ value must be chosen in a way that corresponds to a change of more than a single molecule for the rare species; in fact, for rare species, it should always be a positive integer number of molecules.
- the concentrations of G6P and NADP in the cycle vary with 10% Gaussian noise around the constant values of 0.001 mM and 0.0026 mM, respectively. This signal-to-noise ratio aims to reflect metabolic conditions inside the cell.

The search for optimal kinetic constants was performed using a PC with AMD Ryzen 7 3800X 8-Core Processor, 3900 MHz, RAM 32 GB. Code was written in $C\#$ 8.0. One search epoch simulating one hour for 50 different tables of kinetic constants using all 8 logic cores, took approximately 7 hours.

### Model validation based on the use of experimental data

G6P dehydrogenase is the enzyme that catalyzes the first reaction of the pathway^34^. Therefore, it is the enzyme that controls the starting velocity of the pathway. This enzyme is strongly inhibited by NADPH^35^. Drugs aimed at reducing the intensity of the reaction mainly focus on reducing the activity of this enzyme, which leads to a reduction in the velocity of the entire pathway^27^. However, clinical results indicate that inhibiting this enzyme is not an effective therapeutic approach^26^. For this reason, data obtained from the study of knockdown expression of the 6-phosphogluconate dehydrogenase (PGD) enzyme were selected for model validation^26^.

## Supplementary Information

Supplementary Information.

## Data Availability

The dataset supporting the conclusions of this article is available in the GitHub repository, https://github.com/UTP-WTIiE/PPPQueueingTheory.
