# Supplementary material for: Queueing theory model of pentose phosphate pathway
Source: Sci Rep. 2022 Mar 17;12:4601. doi: 10.1038/s41598-022-08463-y (PMC8930976; doi:10.1038/s41598-022-08463-y)
Supplement: Supplementary file 1 — Supplementary Information. [file 41598_2022_8463_MOESM1_ESM.pdf]

Queueing theory model of pentose phosphate pathway

Sylwester M. Kloska[1,*], Krzysztof Pałczyński[2], Tomasz Marciniak[2], Tomasz Talaśka[2], Marissa Nitz[3], Beata J. Wysocki[4], Paul Davis[4] and Tadeusz A. Wysocki[2,3,*]

[1]Nicolaus Copernicus University Ludwik Rydygier Collegium Medicum, Faculty of Medicine, Bydgoszcz, 85-094, Poland; [2] Bydgoszcz University of Science and Technology, Faculty of Telecommunications, Computer Science and Electrical Engineering, Bydgoszcz, 85-796, Poland; [3]University of Nebraska-Lincoln, Department of Electrical and Computer Engineering, Omaha, NE 68182, USA; [4]University of Nebraska at Omaha, Department of Biology, Omaha, NE 68182, USA

Corresponding author e-mail: 503013@stud.umk.pl; twysocki2@unl.edu

Biochemical data, equations

Metabolite	Metabolite (short)	Concentration [M]	Concentration [mM]
Ribulose-5-P	Ru5P	$1.2*10^{-5}$	0.012
6-P-gluconolactone	PGL	$5*10^{-9}$	$5*10^{-6}$
Glucose-6-P	G6P	$2.6*10^{-6}$	0.0026
ADP	ADP	$7*10^{-4}$	0.7
ATP	ATP	$0.3*10^{-5}$	0.003
$NADP^+$	NADP	$1*10^{-6}$	0.001
NADPH	NADPH	$2*10^{-7}$	0.0002
CO_2	CO2	$1*10^{-6}$	0.001
6-P-gluconate	6PG	$1.8*10^{-5}$	0.018
Ribose-5-P	R5P	$9*10^{-6}$	0.009
Xylulose-5-P	X5P	$1.8*10^{-5}$	0.018
Sedoheptulose-7-P	S7P	$6.8*10^{-5}$	0.068
Glyceraldehyde-3-P	G3P	$2.34*10^{-6}$	0.00234
Erythrose-4-P	E4P	$4*10^{-6}$	0.004
Fructose-6-P	F6P	$8.3*10^{-5}$	0.083

The unit of reaction speed is [µm/min].

1. $G6P + NADP^+ \rightarrow PGL + NADPH + H^+$
   Enzyme: G6PDH

$$V1 = \frac{V_{1F}[NADP][G6P]}{DENOM}$$

$$DENOM = K_{i(NADP)}K_{(G6P)} + K_{(G6P)}[NADP] + K_{(NADP)}[G6P] + [G6P][NADP] + \frac{K_{(G6P)}K_{i(NADP)}}{K_{i(NADPH)}}[NADPH] + \frac{K_{(NADP)}}{K_{i(NADPH)}}[G6P][NADPH]$$

   $V_{1F} = 5.9*10^{-9}$
   $K_{(NADP)} = 4.8*10^{-6}$ M
   $K_{(G6P)} = 3.6*10^{-5}$ M
   $K_{i(NADP)} = 9*10^{-6}$ M
   $K_{i(NADPH)} = 1.1*10^{-6}$ M
2. $PGL + H_2O \rightarrow 6PG + H^+$

Enzyme: 6-gluconolactonase

$$V2 = \frac{V_{2F}\frac{[PGL][H_2O]}{K_{(PGL)} * K_{(H_2O)}} - V_{2R}\frac{[6PG][H^+]}{K_{(6PG)} * K_{(H^+)}}}{\left(1 + \frac{[PGL]}{K_{(PGL)}} + \frac{[6PG]}{K_{(6PG)}}\right) * (1 + \frac{[H_2O]}{K_{(H_2O)}} + \frac{[H^+]}{K_{(H^+)}})}$$

V_{2F}= 5.9*10^{-9}

V_{2R}= 1.232*10^{-12}

$K_{(PGL)}$=8*10^{-5} M

$K_{(6PG)}$=8*10^{-5} M (assumed equal to $K_{(PGL)}$)

3. 6PG + $NADP^+$ → Ru5P + NADPH + H^+ + CO_2
   Enzyme: 6PG dehydrogenase (PGD)

$$V3 = \frac{NUM}{DENOM}$$

$$NUM = V_{3F}[6PG][NADP^+] - (\frac{V_{3R}}{V_{3F}})(\frac{K_{i(NADP)}K_{(6PG)}}{K_{(CO_2)}K_{i(Ru5P)}K_{i(NADPH)}})[CO_2][Ru5P][NADPH]$$

$$\begin{aligned} DENOM &= K_{i(NADP)}K_{(6PG)} + K_{(6PG)}[NADP] + K_{(NADP)}[6PG] + [NADP][6PG] \\ &+ \frac{K_{i(NADP)}K_{(6PG)}K_{(Ru5P)}}{K_{(CO_2)}K_{i(Ru5P)}}[CO_2] + \frac{K_{i(NADP)}K_{(6PG)}}{K_{i(NADPH)}K_{(CO_2)}K_{i(Ru5P)}}[CO_2][Ru5P][NADPH] \\ &+ \frac{K_{(6PG)}K_{(Ru5P)}}{K_{i(6PG)}K_{(CO_2)}K_{i(Ru5P)}}[NADP][6PG][CO_2] + \frac{K_{i(NADP)}K_{(6PG)}}{K_{i(Ru5P)}K_{i(NADPH)}}[Ru5P][NADPH] \\ &+ \frac{K_{(6PG)}K_{(Ru5P)}}{K_{i(Ru5P)}K_{(CO_2)}}[NADP][CO_2] + \frac{K_{(NADP)}}{K_{i(Ru5P)}K_{i(NADPH)}}[6PG][NADPH][Ru5P] \\ &+ \frac{K_{(6PG)}K_{i(NADP)}K_{(NADPH)}}{K_{(CO_2)}K_{i(Ru5P)}K_{i(NADPH)}}[Ru5P][CO_2] + \frac{K_{i(NADP)}K_{(6PG)}}{K_{i(NADPH)}}[NADPH] \\ &+ \frac{K_{(NADP)}}{K_{i(NADPH)}}[6PG][NADPH] + \frac{K_{i(NADP)}K_{(6PG)}K_{(Ru5P)}}{K_{(CO_2)}K_{i(Ru5P)}K_{i(NADPH)}}[NADPH][CO_2] \\ &+ \frac{K_{(NADPH)}K_{(6PG)}K_{i(CO_2)}}{K_{i(6PG)}K_{(CO_2)}K_{i(Ru5P)}K_{i(NADPH)}}[6PG][NADP][Ru5P] \\ &+ \frac{K_{(6PG)}K_{(NADPH)}}{K_{(CO_2)}K_{i(Ru5P)}K_{i(NADPH)}}[NADP][CO_2][Ru5P] \\ &+ (K_{(6PG)}K_{(NADPH)}K_{i(6PG)}K_{(CO_2)}K_{i(Ru5P)}K_{i(NADPH)})[NADP][6PG][CO_2][Ru5P] \\ &+ \frac{K_{(NADP)}}{K_{i(CO_2)}K_{i(Ru5P)}K_{i(NADPH)}}[6PG][CO_2][Ru5P][NADPH] \end{aligned}$$

V_{3F}=4.93*10^{-9}

V_{3R}=1.064*10^{-16}

$K_{(NADP)}$=1.35*10^{-5} M
$K_{i(NADP)}$=4.8*10^{-6} M
$K_{i(NADPH)}$=5.1*10^{-6} M
$K_{(6PG)}$=2.92*10^{-5} M
$K_{(CO2)}$=3.4*10^{-2} M
$K_{(Ru5P)}$=2*10^{-5} M
$K_{(NADPH)}$=2.2*10^{-7} M
K_{eq}=66
$K_{i(6PG)}$=2.176*10^{-3} M
$K_{i(CO2)}$=1.387*10^{-5} M
$K_{i(Ru5P)}$=4.488*10^{-11} M

Note: Both reaction 4A and 4B share the same pool of Ru5P concentration.

4. A) Ru5P → R5P
   enzyme: Ribose-5-phosphate isomerase

$$V4A = \frac{V_{4AF}\frac{[Ru5P]}{K_{Ru5P}} - V_{4AR}\frac{[R5P]}{K_{R5P}}}{(1 + \frac{[Ru5P]}{K_{Ru5P}} + \frac{[R5P]}{K_{R5P}})}$$

V_{4AF}=5.9*10^{-9}
V_{4AR}=1.1225*10^{-8}
$K_{(Ru5P)}$=7.8*10^{-4} M
$K_{(R5P)}$=2.2*10^{-3} M

4. B) Ru5P → X5P
   Enzyme: Ribulose 5-Phosphate 3-Epimerase

$$V4B = \frac{V_{4BF}\frac{[Ru5P]}{K_{Ru5P}} - V_{4BR}\frac{[X5P]}{K_{X5P}}}{(1 + \frac{[Ru5P]}{K_{Ru5P}} + \frac{[X5P]}{K_{X5P}})}$$

V_{4BF}=5.9*10^{-9}
V_{4BR}=8.48*10^{-9}
$K_{(Ru5P)}$=1.9*10^{-4} M
$K_{(X5P)}$=5*10^{-4} M

5. X5P + R5P → G3P + S7P
   Enzyme: transketolase

$$V5 = \frac{NUM5}{DENOM5}$$

$$NUM5 = K_5[R5P][X5P] + K_2[F6P][R5P] - K_3[S7P][G3P] - K_4[S7P][E4P]$$

$$DENOM5 = K_m\left(1+\frac{[G6P]}{K_{i(R5P)}}\right)\left(1+\frac{[G6P]}{K_{i(X5P)}}\right)+K_{R5P}[R5P]\left(1+\frac{[G6P]}{K_{i(R5P)}}\right) + K_{X5P}[X5P]\left(1+\frac{[G6P]}{K_{i(X5P)}}\right)$$

$$K_m = K_5[S7P]+K_6[G3P]+K_7[F6P]+K_{10}[E4P]+K_{12}[S7P][G3P] + K_{13}[S7P][E4P]+K_{14}[R5P][X5P]+K_{18}[G3P][F6P] + K_{19}[F6P][E4P]$$

$$K_{R5P} = K_8 + K_{11}[S7P] + K_{15}[F6P]$$

$$K_{X5P} = K_9 + K_{16}[G3P] + K_{17}[E4P]$$

K_1=6*10^{-7} M
K_2=1.1*10^{-12} M
K_3=1.006*10^{-8} M
K_4=9.9*10^{-13} M
K_5=1.09*10^{-3} M
K_6=3.2*10^{-6} M
K_7=1.55*10^{-2} M
K_8=3.8*10^{-4} M
K_9=1.548*10^{-6} M
K_{10}=3.8*10^{-4} M
K_{11}=1.267 M
K_{12}=6.05 M
K_{13}=10^{-5} M
K_{14}=1 M
K_{15}=10^{-5} M
K_{16}=0.0086 M
K_{17}=1 M
K_{18}=86.4 M
K_{19}=8.64 M
K_{20}=5.9*10^{-9} M
K_{21}=2.2*10^{-12} M
K_{22}=3.802*10^{-10} M
K_{23}=5.9*10^{-13}
$K_{i(R5P)}$=0.82 mM
$K_{i(X5P)}$=3.6 mM

6. X5P + E4P → G3P + F6P
   Enzyme: transketolase

$$V6 = \frac{NUM6}{DENOM6}$$

$$NUM6 = K_{20}[X5P][E4P] + K_{21}[S7P][E4P] - K_{22}[F6P][G3P] - K_{23}[F6P][R5P]$$

$$DENOM6 = K_m\left(1 + \frac{[G6P]}{K_{i(R5P)}}\right)\left(1 + \frac{[G6P]}{K_{i(X5P)}}\right) + K_{R5P}[R5P]\left(1 + \frac{[G6P]}{K_{i(R5P)}}\right) + K_{X5P}[X5P]\left(1 + \frac{[G6P]}{K_{i(X5P)}}\right)$$

$$K_m = K_5[S7P] + K_6[G3P] + K_7[F6P] + K_{10}[E4P] + K_{12}[S7P][G3P] + K_{13}[S7P][E4P] + K_{14}[R5P][X5P] + K_{18}[G3P][F6P] + K_{19}[F6P][E4P]$$

$$K_{R5P} = K_8 + K_{11}[S7P] + K_{15}[F6P]$$

$$K_{X5P} = K_9 + K_{16}[G3P] + K_{17}[E4P]$$

K_1=6*10^{-7} M
K_2=1.1*10^{-12} M
K_3=1.006*10^{-8} M
K_4=9.9*10^{-13} M
K_5=1.09*10^{-3} M
K_6=3.2*10^{-6} M
K_7=1.55*10^{-2} M
K_8=3.8*10^{-4} M
K_9=1.548*10^{-6} M
K_{10}=3.8*10^{-4} M
K_{11}=1.267 M
K_{12}=6.05 M
K_{13}=10^{-5} M
K_{14}=1 M
K_{15}=10^{-5} M
K_{16}=0.0086 M
K_{17}=1 M
K_{18}=86.4 M
K_{19}=8.64 M
K_{20}=5.9*10^{-9} M
K_{21}=2.2*10^{-12} M
K_{22}=3.802*10^{-10} M
K_{23}=5.9*10^{-13}
$K_{i(R5P)}$=0.82 mM
$K_{i(X5P)}$=3.6 mM

7. S7P + G3P → E4P + F6P
   Enzyme: transaldolase

$$V7 = \frac{NUM7}{DENOM7}$$

$$NUM7 = V_{7F}[[S7P][G3P] - \frac{V_{7R}}{V7_F}\frac{K_{i(S7P)}K_{(G3P)}}{K_{(F6P)}K_{i(E4P)}}[E4P][F6P]]$$

$$DENOM7 = K_{(G3P)}[S7P] + K_{(S7P)}[G3P] + [S7P][G3P] + \frac{K_{i(S7P)}K_{G3P}}{K_{i(E4P)}}[E4P] + \frac{K_{i(S7P)}K_{G3P}}{K_{i(E4P)}K_{(F6P)}}[F6P] + \frac{K_{(G3P)}}{K_{i(E4P)}}[S7P][E4P] + \frac{K_{(S7P)}}{K_{i(F6P)}}[G3P][F6P]$$

$V_{7F}=5.9*10^{-9}$

$V_{7R}=1.776*10^{-9}$

$K_{(S7P)}=1.8*10^{-4}$ M

$K_{(G3P)}=2.2*10^{-4}$ M

$K_{(E4P)}=7*10^{-6}$ M

$K_{(F6P)}=2*10^{-4}$ M

$K_{i(S7P)}= 1.8*10^{-4}$ M

$K_{i(F6P)}=2*10^{-4}$ M

$K_{i(E4P)}=7*10^{-6}$ M

PPP Pseudocode:

1. chr1 <- input first chromosome
2. chr2 <- input second chromosome
3. mut_chance <- input mutation chance
4. mut_amp <- input mutation amplitude
5. constraints <- input table of constraints forcing reaction of corresponding index to have value between minimum and maximum value stored in the table
6. p <- input vector of initial products in the simulation
7. cc1 <- divide chromosome ch1 to subsets, where each contains all constants required for calculating one reaction
8. cc2 <- divide chromosome ch2 to subsets, where each contains all constants required for calculating one reaction
9. cc3 <- create empty set of subsets of genes
10. for $i \in < 0; |cc1|] \cap N^{+}$:
    a. c1 = cc1[i]
    b. c2 = cc2[i]
    c. random_c = pick random number from set of values {0, 1}

d. c3 <- c1 if c == 0 else c2

e. for $j \in < 0; |c1|] \cap N^{+}$:

   i. rand <- generate random value from uniform distribution from 0 to 1

   ii. if rand < mut_chance:

      1. norm_rand <- generate random value from normal distribution

      2. c3[j] = c3[j] * (1 + mut_amp * norm_rand)

      3. c3[j] = |c3[j]|

f. c3_prob <- calculate probability of reaction using c3 and p

g. if c3_prob > constraints[i].min and c3_prob < constraints[i].max:

   i. cc3.append(c3)

   ii. perform next iteration of for loop

h. else:

   i. c3_score <- calculate distance of c3 rate probability to closest limit of constraints

   ii. c1_score <- calculate distance of c1 rate probability to closest limit of constraints. If c1 rate probability is within range, then c1_score = 0

   iii. calculate distance of c2 rate probability to closest limit of constraints. If c2 rate probability is within range, then c2_score = 0

   iv. scores = {(c1, c1_score), (c2, c2_score), (c3, c3_score)}

   v. sort scores by second field ascending

   vi. c1 = scores[0][0]

   vii. c2 = scores[1][0]

   viii. go to step 10. C

11. return cc

Pseudocode of PPP cycle simulation

Procedure simulation (p_start, iter, sec, c, noise, q):

1. p_start ← input table containing masses of products at the beginning of simulation
2. iter ← input number of iterations of the experiment
3. sec ← input how many seconds should one iteration simulate
4. c ← input table of vectors of kinetic constants
5. noise ← input amplitude of gaussian noise
6. records ← create table of size (sec x 13), which stores current amount of each product’s mass at every second of the experiment

7. q ← input size of changed value in queues
8. iterate for $i \in <0; iter) \cap N^{+}$:
    a. copy p_start to p
    b. iterate for $s \in <0; sec\ ) \cap N^{+}$:
        i. records[s] += p / iter
        ii. iterate for $ms \in <0; 1000) \cap N^{+}$:
            1. p = compute_one_timestep(p, c, noise, q)
            2. records[s] = p
9. return records

Procedure compute_one_timestep (p, c, noise, q):

1. p ← input current products vector
2. c ← input kinetic constants of simulation divided into arrays selected for every rate
3. noise ← input amplitude of gaussian noise
4. q ← input size of changed value in queues
5. for $i \in <0; 1000)$
    a. compute_rate1_queue(p, c[0], noise, q)
    b. compute_rate4a_queue(p, c[3], noise, q)
    c. compute_rate4b_queue(p, c[4], noise, q)
    d. compute_rate7_queue(p, c[7], noise, q)
6. compute_rate2_queue(p, c[1], noise, q)
7. compute_rate3_queue(p, c[2], noise, q)
8. compute_rate5_queue(p, c[5], noise, q)
9. compute_rate6_queue(p, c[6], noise, q)

Procedure compute_rate1_queue(p, c, noise, q):

1. p <- input current products vector
2. cc <- input kinetic constants of rate2
3. q ← input size of changed value in queues
4. copy cc to _cc
5. apply gaussian noise to _c of amplitude = n
6. V = (_cc[0] * p[0] / _cc[2] – 0.01 * _cc[1] * p[2] * _cc[3]) / (1 + p[0] / _cc[2] + p[2] / _cc[3])
7. r <- generate random number from uniform distribution from 0 to 1
8. if r < V:

   a. p[2] += q
9. return p

Procedure compute_rate2_queue(p, cc, n, q):

1. p <- input current products vector
2. cc <- input kinetic constants of rate2
3. q ← input size of changed value in queues
4. copy cc to _cc
5. apply gaussian noise to _c of amplitude = n
6. V=(_cc[2]*(p[2]/_cc[0])-(_cc[3]*(p[4]/_cc[1])))/(1+(p[2]/_cc[0])+(p[4]/_cc[1]))
7. r <- generate random number from uniform distribution from 0 to 1
8. if r < V:
   a. p[2] -= q
   b. p[4] += q
9. return

Procedure compute_rate3_queue(p, cc, n, q):

1. p <- input current products vector
2. cc <- input kinetic constants of rate2
3. q ← input size of changed value in queues
4. copy cc to _cc
5. apply gaussian noise to _c of amplitude = n
6. a=(_cc[0]*p[4]*p[1])-((_cc[0]/_cc[1])*((_cc[3]*_cc[5])/(_cc[6]*_cc[12]*_cc[4]))*p[12]*p[5]*p[3])
7. b=(_cc[3]*_cc[5])+(_cc[5]*p[1])+(_cc[2]*p[4])+(p[4]*p[1])+((_cc[3]*_cc[5]*_cc[7])/(_cc[6]*_cc[12]))*p[12]+(((_cc[3]*_cc[5])/(_cc[4]*_cc[6]*_cc[12]))*p[12]*p[5]*p[3])+(((_cc[5]*_cc[7])/(_cc[10]*_cc[6]*_cc[12]))*p[1]*p[4]*p[12])+(((_cc[3]*_cc[5])/(_cc[12]*_cc[4]))*p[5]*p[3])+(((_cc[5]*_cc[7])/(_cc[12]*_cc[6]))*p[1]*p[12])+((_cc[2]/(_cc[12]*_cc[4]))*p[4]*p[3]*p[5])+(((_cc[5]*_cc[3]*_cc[8])/(_cc[6]*_cc[12]*_cc[4]))*p[5]*p[12])+(((_cc[3]*_cc[5])/_cc[4])*p[3])+((_cc[2]/_cc[4])*p[4]*p[3])+(((_cc[3]*_cc[5]*_cc[7])/(_cc[6]*_cc[12]*_cc[4]))*p[12]*p[3])+(((_cc[8]*_cc[5]*_cc[11])/(_cc[10]*_cc[6]*_cc[12]*_cc[4]))*p[4]*p[1]*p[5])+(((_cc[5]*_cc[8])/(_cc[6]*_cc[12]*_cc[4]))*p[1]*p[12]*p[5])+(_cc[5]*_cc[8]*_cc[10]*_cc[6]*_cc[12]*_cc[4])*(p[1]*p[4]*p[12]*p[5])+((_cc[2]/(_cc[11]*_cc[12]*_cc[4]))*p[4]*p[12]*p[5]*p[3])
8. V=a/b

9. r <- generate random number from uniform distribution from 0 to 1
10. if r < V:
    a. p[4] -= q
    b. p[5] += q
11. return p

Procedure compute_rate4a_queue(p, cc, n, q):

1. p <- input current products vector
2. cc <- input kinetic constants of rate2
3. q ← input size of changed value in queues
4. copy cc to _cc
5. apply gaussian noise to _c of amplitude = n
6. V=(_cc[2]*(p[5]/_cc[0])-(_cc[3]*(p[6]/_cc[1])))/(1+(p[5]/_cc[0])+(p[6]/_cc[1]))
7. r <- generate random number from uniform distribution from 0 to 1
8. if r < |V|:
    a. sign <- 1 if r > 0 else -1
    b. p[5] -= q * sign
    c. p[6] += q * sign
9. return p

Procedure compute_rate4b_queue(p, cc, n, q):

1. p <- input current products vector
2. cc <- input kinetic constants of rate2
3. q ← input size of changed value in queues
4. copy cc to _cc
5. apply gaussian noise to _c of amplitude = n
6. V=(_cc[2]*(p[5]/_cc[0])-(_cc[3]*(p[7]/_cc[1])))/(1+(p[5]/_cc[0])+(p[7]/_cc[1]))
7. r <- generate random number from uniform distribution from 0 to 1
8. if r < |V|:
    a. sign <- 1 if r > 0 else -1
    b. p[5] -= q * sign
    c. p[7] += q * sign
9. return p

Procedure compute_rate5_queue(p, cc, n, q):

1. p <- input current products vector
2. cc <- input kinetic constants of rate2
3. q ← input size of changed value in queues
4. copy cc to _cc
5. apply gaussian noise to _c of amplitude = n
6. b1=_cc[4]*p[9]+_cc[5]*p[8]+_cc[6]*p[11]+_cc[9]*p[10]+_cc[11]*p[9]*p[8]+_cc[12]*p[9]*p[10]+_cc[13]*p[6]*p[7]+_cc[17]*p[8]*p[11]+_cc[18]*p[11]*p[10] #_cc[1]_cc[3]
7. b2=_cc[7]+_cc[10]*p[9]+_cc[14]*p[11] #_cc[1](_cc[8]5P)
8. b3=_cc[8]+_cc[15]*p[8]+_cc[16]*p[10] #_cc[1](_cc[14]5P)
9. a=(_cc[4]*p[6]*p[7])+(_cc[1]*p[11]*p[6])-(_cc[2]*p[9]*p[8])-(_cc[3]*p[9]*p[10]) #_cc[4]_cc[11]_cc[3]5
10. b=(b1*(1+(p[0]/_cc[22]))*(1+(p[0]/_cc[23]))+(b2*p[6]*(1+(p[0]/_cc[22])))+(b3*p[7]*(1+(p[0]/_cc[23]))) #p[7]p[11]_cc[4]_cc[5]_cc[3]5
11. V=a/b
12. r <- generate random number from uniform distribution from 0 to 1
13. if r < |V|:
    a. sign <- 1 if r > 0 else -1
    b. p[7] -= q * sign
    c. p[6] += q * sign
14. return p

Procedure compute_rate6_queue(p, cc, n, q):

1. p <- input current products vector
2. cc <- input kinetic constants of rate2
3. q ← input size of changed value in queues
4. copy cc to _cc
5. apply gaussian noise to _c of amplitude = n
6. b1=_cc[4]*p[9]+_cc[5]*p[8]+_cc[6]*p[11]+_cc[9]*p[10]+_cc[11]*p[9]*p[8]+_cc[12]*p[9]*p[10]+_cc[13]*p[6]*p[7]+(_cc[17]*p[8]*p[11])+(_cc[18]*p[11]*p[10])
7. b2=_cc[7]+_cc[10]*p[9]+_cc[14]*p[11]
8. b3=_cc[8]+_cc[15]*p[8]+_cc[16]*p[10]

9. a=(_cc[19]*p[7]*p[10])+(_cc[20]*p[9]*p[10])-(_cc[21]*p[11]*p[8])-(_cc[22]*p[11]*p[6])
10. b=(b1*(1+(p[0]/_cc[23]))*(1+(p[0]/_cc[24]))+(b2*p[6]*(1+(p[0]/_cc[23])))+(b3*p[7](1+(p[0]/_cc[24])))
11. V6=a/b
12. r <- generate random number from uniform distribution from 0 to 1
13. if r < |V|:
    a. sign <- 1 if r > 0 else -1
    b. p[7] -= q * sign
    c. p[10] -= q * sign
    d. p[8] += q * sign
    e. p[11] += q * sign
14. return p

Procedure compute_rate7_queue(p, cc, n, q):

1. p <- input current products vector
2. cc <- input kinetic constants of rate2
3. q ← input size of changed value in queues
4. copy cc to _cc
5. apply gaussian noise to _c of amplitude = n
6. a=_cc[0]*((p[9]*p[8])-((_cc[0]/_cc[1])*((_cc[6]*_cc[3])/(_cc[5]*_cc[8]))*(p[10]*p[11])))
   #_cc[7]U_cc[6]7
7. b=(_cc[3]*p[9])+(_cc[2]*p[8])+p[9]*p[8]+(((_cc[6]*_cc[3])/_cc[8])*p[10])+(((_cc[6]*_cc[3])/(_cc[8]*_cc[5]))*p[11])+((_cc[3]/_cc[8])*p[9]*p[10])+((_cc[2]/_cc[7])*p[8]*p[11])
   #_cc[1]p[9]_cc[7]_cc[8]_cc[6]7
8. V=a/b
9. r <- generate random number from uniform distribution from 0 to 1
10. if r < |V|:
    a. sign <- 1 if r > 0 else -1
    b. p[9] -= q * sign
    c. p[8] -= q * sign
    d. p[10] += q * sign
    e. p[11] += q * sign
11. return p
